# The energy-growth nexus revisited: the role of financial development, institutions, government expenditure and trade openness

**DOI:** 10.1016/j.heliyon.2020.e04369

**Published:** 2020-07-14

**Authors:** Hoang Phong Le

**Affiliations:** School of Public Finance, University of Economics Ho Chi Minh City, 59C Nguyen Dinh Chieu, District 3, Ho Chi Minh City, Viet Nam

**Keywords:** Economic growth, Energy use, Financial development, Institutions, Government expenditures, Trade openness, Emerging market and developing economies, EMDEs, Public economics, Economic development, Macroeconomics, Energy economics, Economics

## Abstract

Although the energy-growth nexus has been widely investigated in the last several decades, there are still vivid debates in the energy economics field. This study evaluates the link between energy consumption and economic growth with the thorough assessment of the roles of institutional quality, government expenditure, financial development and trade openness in 46 Emerging Market and Developing Economies (EMDEs) from 1990 to 2014. By employing appropriate panel econometric techniques, cross-sectional dependence and slope heterogeneity are controlled, which helps explore the unbiased long-run effects of the determinants of economic growth as well as scrutinize the dynamic relationship among variables. The findings demonstrate that energy usage, gross fixed capital formation, government expenditure, financial development and trade openness positively and significantly impact the economic growth in the studied EMDEs. Moreover, Dumitrescu and Hurlin causality tests affirm the occurrence of feedback hypothesis in the connection between energy consumption and other variables including economic growth. Thus, it implies that energy consumption and economic growth are interdependent, which forms a basis for policy-makers to design effective energy and environmental policies. Toward the sustainable development goal, the author recommends the governments of EMDEs to contemplate the importance of finance-governance-trade relationship to economic growth alongside the implementation of energy-efficient policies.

## 1 Introduction

The vast majority of countries depend on energy industry in their development processes, and the world's demand for energy is higher and higher (Le and Sarkodie, 2020). According to British Petroleum (2018), primary energy consumption increased by 2.2% in 2017, which marks its fastest growth rate since 2013. The incremental energy consumption for economic activities raises a big issue of escalating environmental degradation (Dong et al., 2018; McConnell et al., 2018; Phong et al., 2018). Carbon dioxide (CO_2_), a main component of greenhouse gas, was emitted to the air at about 33,472.0 million metric tons in 2014, increasing dramatically from nearly 21,571.7 metric tons in 1990 with the annual growth rate of roughly 1.8% (BP, 2017). The emission of CO_2_ has been so large that in 2050 it may reach its peak in at least the last 50 million years (Foster et al., 2017). Obviously, the worsened environmental quality has contributed to the improvement of environmental protection awareness which focuses on the role of efficient energy use in sustainable economic development (Phong, 2019; Le and Ozturk, 2020).

The relationship between energy consumption and economic growth has been widely studied in recent decades, thus being one of the most important topics in energy economics literature (see Kraft and Kraft, 1978; Stern, 1993; Stern, 2000; Ozturk, 2010; Belke et al., 2011; Magazzino, 2015; Shahbaz et al., 2011, 2013, 2017, 2018). Nevertheless, the findings on the causation between energy consumption and economic growth are not similar, which presumably stems from the selections of countries, time and econometric methods (Le and Van, 2020). In order to thoroughly assess the effects of energy consumption on economic growth, the combination of variables in a multivariate function is of vital importance. This can avoid inconsistent and bias estimates (Lütkepohl, 1982; Smyth and Narayan, 2015).

This study focuses on the Emerging Market and Developing Economies (EMDEs) because they are the open and dynamic economies that have rapidly integrated into the global economy via commerce and investment (Gruss et al., 2018). Over the past few decades, EMDEs have increasingly contributed to the global output and consumption, occupying around 70% in the period 2000–2015 (Gruss et al., 2018). However, EMDEs face serious challenges as they have relatively low institutional quality combined with low financial development level as well as energy security and weak environmental protection standards (Ghosh, 2010; Saidi and Mbarek, 2017; Slesman et al., 2019; Le and Ozturk, 2020). Hence, the detailed examination of factors influencing the economic growth of EMDEs is crucial for their stable and sustainable development. As a result, the author employs panel data econometric techniques for multivariate model based on the extended Cobb-Douglas production function.

This article contributes to the existing literature in three aspects. First, in order to avoid the omission of variables leading to unreliable findings, this study extends the production function using energy consumption, gross fixed capital formation, institutions, government expenditure, financial development, and trade openness as explanatory variables to enrich the current literature for Emerging Market and Developing Economies, which has not been conducted by any prior research. Second, regarding the measurement of variables, the author uses the composite index of corruption, law and order and bureaucracy quality to quantify the “institutions” variable, which can better represent the quality of government governance than each separate criterion. Moreover, this paper employs 03 individual components as well as the overall index as proxies for financial development in order to evaluate its effects on economic growth in the incorporate with the other variables and check for robustness of the results. Third, concerning the possibility of cross-sectional dependence and heterogeneity within cross countries, this research applies methods that deal well with the aforementioned issues and are more powerful in analyzing the impacts of input factors on economic growth. The empirical results can provide more policy implications when using parameter estimating tools and causality procedure. The estimation of parameters is conducted by the Augmented Mean Group (AMG) estimator (Eberhardt and Bond, 2009; Eberhardt and Teal, 2010), while the Mean Group (MG) (Pesaran and Smith, 1995) and the Common Correlated Effects Mean Group (CCEMG) (Pesaran, 2006) estimators are also employed for robustness check.

The remaining content of this article is structure as follows: Section 2 provides important review of the current literature; Section 3 describes the Model, Data, and Methodology; Section 4 displays the empirical results and explanations to the findings; Section 5 demonstrates notable summaries of this paper as well as policy implications.

## 2 Literature review

The relationship between energy consumption and economic growth can be explained by 04 main hypotheses including growth hypothesis, conservation hypothesis, feedback hypothesis, and neutrality hypothesis (Apergis and Payne, 2009b; Ozturk, 2010; Apergis and Tang, 2013). The growth hypothesis states that higher energy consumption leads to economic growth. Meanwhile, the conservation hypothesis assumes that the rise of income level boosts energy consumption, and the feedback hypothesis proposes two-way causation between these variables. On the contrary, the neutrality hypothesis supposes no or insignificant connection between energy consumption and economic growth. Table 1 below displays some empirical studies and the hypotheses that they supported.

**Table 1:** Brief review of energy-growth studies and the supported hypotheses.

Author(s)	Countries	Period	Methodology	Results
Asafu-Adjaye (2000)	Indonesia, India, Thailand, Philippine	(a) 1973–1995 (Indonesia, India) (b) 1971–1995 (Thailand, Philippine)	Cointegration and Granger causality based on ECM	(a) Growth hypothesis (Indonesia, India) (b) Feedback hypothesis (Thailand, Philippine)
Wolde-Rufael (2004)	Shanghai	1952–1999	Toda and Yamamoto Granger causality	Growth hypothesis
Lee (2005)	18 developing countries	1975–2001	FMOLS; Panel causality tests	Growth hypothesis
Lee (2006)	11 developed countries	1960–2001	Granger causality test	Neutrality hypothesis
Mahadevan and Asafu-Adjaye (2007)	20 developed and developing countries	1971–2002	Pedroni Panel cointegration, panel VECM	Feedback hypothesis
Huang et al. (2008)	82 countries	1972–2002	System GMM	(a) Conservation hypothesis (63 middle & high income countries) (b) Neutrality hypothesis (19 low income countries)
Apergis and Payne (2009b)	6 Central American countries	1980–2004	Pedroni Panel cointegration, error correction model	Growth hypothesis
Payne (2009)	US	1949–2006	Toda-Yamamoto causality tests	Neutrality hypothesis
Ozturk et al. (2010)	51 countries	1971–2005	Pedroni panel cointegration; FMOLS, DOLS; and panel causality tests	(a) Feedback hypothesis (middle income countries) (b) Conservation hypothesis (low income countries)
Belke et al. (2011)	25 OECD countries	1981–2007	Panel cointegration; VECM; Granger causality	Feedback hypothesis
Tugcu et al. (2012)	G7	1980–2009	ARDL approach; Hatemi-J causality test	Feedback hypothesis
Shahbaz et al. (2013)	China	1971–2011	ARDL, Johansen and Juselies test; VECM granger causality	Growth hypothesis
Al-Mulali et al. (2014)	18 Latin American countries	1980–2010	DOLS, VECM	Feedback hypothesis
Kumar et al. (2015a)	South Africa	1971–2011	ARDL; Bayer and Hanck cointegration; Toda and Yamamoto Granger causality	Growth hypothesis
Kumar et al. (2015b)	Gibraltar	1996–2012	ARDL; Toda and Yamamoto Granger causality	Growth hypothesis
Destek (2016)	Newly industrialized countries	1971–2011	ARDL; Hatemi-J causality	Neutrality hypothesis (Brazil, Malaysia)
Furuoka (2017)	Estonia, Latvia and Lithuania	1992–2011	Granger causality, Dumitrescu-Hurlin panel causality	Conservation hypothesis.
Mbarek et al. (2018)	Tunisia	1990–2015	Granger causality test and VECM model	Growth hypothesis
Rasoulinezhad and Saboori (2018)	Commonwealth of Independent States region	1992–2015	DOLS and FMOLS; Pedroni and Kao panel cointegration; Panel Granger causality; Dumitrescu-Hurlin panel causality test	Feedback hypothesis
Saidi et al. (2019)	MENA countries	1986–2015	Pedroni, Westerlund, and Westerlund and Edgerton cointegration; FMOLS and DOLS; Panel Error Correction Models	Feedback hypothesis
Zafar et al. (2019)	APEC countries	1990–2015	CUP-BC, CUP-FM; Heterogeneous panel causality	Feedback hypothesis
Le and Bao (2020)	16 Latin America and Caribbean countries	1990–2014	AMG, CCEMG and MG estimators; Dumitrescu-Hurlin panel causality	Feedback hypothesis
Le and Van (2020)	20 African countries	1990–2014	AMG, CCEMG and MG estimators; Dumitrescu-Hurlin panel causality	Feedback hypothesis

Notes: AMG: Augmented mean group; ARDL: autoregressive distributed lag; CCEMG: Common correlated effects mean group; CUP-BC: Continuously updated bias-corrected; CUP-FM: Continuously updated fully modified ordinary least square; DOLS: Dynamic ordinary least squares; ECM: Error correction model; FMOLS: Fully modified OLS; GMM: generalized methods of moments; MG: Mean group; VECM: Vector error correction model.

From the aforesaid studies in particular (see Table 1) and the literature on energy economics in general, it can be witnessed that different authors employed different methods and validated different hypotheses. Despite the lack of consensus on explaining the link between energy consumption and economic growth, the existing literature points out that energy is an essential input factor of economic growth besides capital and labor (Kraft and Kraft, 1978; Apergis and Payne, 2009a, b; Arbex and Perobelli, 2010).

With the development of researches on energy economics, many studies have employed the extended production function in which some variables such as financial development and trade openness are added to the right-hand side of the equation besides capital, labor and energy (see Smyth and Narayan, 2015). The roles of financial development in economic growth are explained by 02 transmission channels: distribution and accumulation (Schumpeter, 1911; Shahbaz et al., 2013; Ruiz, 2018). Particularly, through the distribution channel, financial development enhances the effectiveness of resources allocation (Schumpeter, 1911); and through the accumulation channel, financial development facilitates the accumulation of material and human capital (Shahbaz et al., 2013; Ruiz, 2018). Besides financial development, trade openness is a considerable stimulant of growth. The literature on endogenous growth models (Rivera-Batiz and Romer, 1991; Romer, 1994; Barro and Sala-i-Martin, 1997) indicate that trade openness can enhance goods and services transactions, improve efficiency in resources allocation and enhance the total factor productivity by disseminating or transferring technology and knowledge, which in turns facilitates economic growth in the long-run (Frankel and Romer, 1999; Dollar and Kraay, 2004; Chang et al., 2009; Shahbaz, 2012). Furthermore, in order for an economy operates properly, the financial-institutional role of the government is of the essence. The endogenous growth theory of Romer (1986), Lucas (1988) and Barro (1990) provided the mechanisms for government sector to affect not only the output level but also the stable growth rate. Government expenditure on public goods (household subsidies through education, medical and social transfer, environmental protection, research and development and dissemination of knowledge, institution and law) positively impacts economic growth via scale effect (López et al., 2011). According to institutional economics theories, better institutional quality fosters economic activities via the reduction of asymmetric information, transaction costs and risks as well as via the enhancement of market efficiency, asset allocation and property right (Williamson, 1981; Cohen et al., 1983; Ho and Michaely, 1988; La Porta et al., 1997; Fredriksson et al., 2004; Hallerberg and Wolff, 2008; Fosu, 2014).

Shahbaz et al. (2013) included financial development, international trade, capital and energy consumption and found that all the variables positively affected the economic growth of China in the period 1971–2011; also, the Granger causality analysis confirmed the growth hypothesis between energy consumption and economic growth. Kumar et al. (2015a) examined the roles of energy, trade, financial development and capital in explaining the economic growth of South Africa from 1971 to 2011 by ARDL method. The results demonstrated that energy, capital and trade openness promoted economic growth in both short-run and long-run, while financial development worsened it. The Granger causality test indicated one-way causation from energy to growth, thus verifying the growth hypothesis. Roubaud and Shahbaz (2018) employed the extended production function to examine the link between electricity consumption and economic growth at aggregate and industry levels in Pakistan from 1972 to 2014, showing that financial development was an important stimulant of electricity consumption and economic growth. Besides, causality analysis acknowledged the presence of feedback hypothesis between electricity consumption and economic growth. Zafar et al. (2019) studied the impacts of non-renewable and renewable energy consumption in conjunction with capital formation, research and development expenditures and trade openness on the economic growth of Asia-Pacific Economic Cooperation (APEC) countries by utilizing CUP-FMOLS method for long-run estimation of the panel data ranging from 1990 to 2015, which exhibited that higher energy use caused economic growth. Likewise, capital formation, research and development expenditures and trade openness positively influenced economic growth, thus supporting the feedback hypothesis (i.e. two-way causality between energy consumption and economic growth). Recently, some studies have considered the roles of institutions and government expenditure as additional factors of growth models used in energy-growth nexus researches. For instance, Saidi et al. (2019) investigated the role of institutions in the energy-growth nexus in MENA countries from 1986 to 2015. They employed panel cointegration tests and detected two-way causality between renewable energy usage and economic growth as well as between institutions (except law and order) and economic growth. Le and Bao (2020) extended the Cobb-Douglas production function using data from 16 Latin American and Caribbean countries. They utilized second-generation econometric techniques for heterogeneous panel and found that government expenditure, institutional quality, renewable and nonrenewable energy consumption, capital, trade openness and financial development positively impact the economic growth of the selected Latin America and Caribbean countries. Besides, they validated the feedback hypothesis and documented the bidirectional causation between energy usage and economic growth. Following this topic, Le and Van (2020) observed the positive effects of energy use components, capital, government expenditure and trade openness on economic growth in 20 African countries.

In general, given that EMDEs face challenges such as relatively weak institutional quality, low financial development level and environmental protection standards as well as energy security, after carefully assessing the existing literature, this study examines the impacts of energy consumption on the economic growth of EMDEs with the incorporation of capital, government expenditure, institutional quality, financial development and trade openness so as to give some recommendations for sustainable development policies.

## 3 Model, methodology, and data

### 3.1 Model specification

The main objective of this article is to evaluate the effects of energy consumption, government expenditure, institutional quality, financial development and trade openness on the economic growth of Emerging Market and Developing Economies. Hence, we employ the extended Cobb-Douglas production function as follows:

(1) $$Y=AK^{\alpha_{1}}L^{\alpha_{2}}EC^{\alpha_{3}}e^{\mu }$$

where Y is domestic output, A represents technological factor, K denotes capital, L indicates labor, EC stands for energy consumption, and e stands for error. In addition, $\alpha_{1},\alpha_{2},\alpha_{3}$ are respectively the capital, labor and energy consumption elasticities of output.

In Eq. (1), the extended Cobb-Douglas production function has constant returns to scale when it is restricted by the condition $\alpha_{1}+\alpha_{2}+\alpha_{3}=1$. Also, it is assumed that the technological factor can be endogenously determined by the levels of financial and international trade development (Shahbaz, 2012; Shahbaz et al., 2013; Le and Bao, 2020). As a result, the technological factor can be illustrated as follows:

(2) $$A_{t}=\tau FD_{t}^{\varphi_{1}}TO_{t}^{\varphi_{2}}$$

where τ is a time-invariant constant, FD represents financial development, and TO denotes trade openness.

From Eqs. (1) and (2), Eq. (3) is deduced as:

(3) $$Y_{t}=\tau FD_{t}^{\varphi_{1}}TO_{t}^{\varphi_{2}}EC_{t}^{\alpha_{3}}K_{t}^{\alpha_{1}}L_{t}^{1-\alpha_{1}-\alpha_{3}}$$

Then, dividing both sides of Eq. (3) by the population, we have a time-series under per capita terms. Meanwhile, the effect of labor is kept unchanged, and we add the role of government in terms of government expenditure and institutional quality as control variables (see Le and Bao, 2020; Le and Sarkodie, 2020; Le and Van, 2020). Now, we transform Eq. (3) with the control variables into log-linear form:

(4) $$lnGDP_{it}=\alpha_{it}+\beta_{1}lnGCF_{it}+\beta_{2}lnGC_{it}+\beta_{3}lnQoG_{it}+\beta_{4}lnEC_{it}+\beta_{5}lnFD_{it}+\beta_{6}lnTO_{it}+\varepsilon_{it}\;$$

In Eq. (4), GDP, GCF, GC, QoG, EC, FD, TO respectively are real GDP, gross fixed capital formation, general government final consumption expenditure, institution quality, energy consumption, financial development, and trade openness. All variables are under “per capita” form except institutional quality and financial development index. Additionally, “ln” denotes natural logarithm, i represents each country in the panel data (i=1, 2,…, N), t indicates the time period (1990–2014), $\;{\text{ε}}_{\text{it}}$ is the error term and the coefficients ${\text{β}}_{1},\;{\text{β}}_{2},{\text{β}}_{3},{\text{β}}_{4},{\text{β}}_{5},\;and\;{\text{β}}_{6}$ reflect the impacts of the regressors on the dependent variable.

### 3.2 Econometric methodology

#### 3.2.1 Cross-sectional dependence test

Different countries may interact with each other through the connections in an economic-social network consisting of activities such as investment, import, export and economic-social integration, which possibly leads to cross-sectional dependence among them. Besides, cross-sectional dependence can result from other factors such as model misspecification and common shocks (Chudik and Pesaran, 2013). If cross-sectional dependence is not controlled, the estimation outcomes can be biased and inconsistent (Breusch and Pagan, 1980; Pesaran, 2004; Phillips and Sul, 2003). This necessitates the detection of cross-sectional dependence in the panel data analysis.

In order to disclose the cross-sectional dependence in panel data, the well-known Langrage multiplier (LM) cross-sectional dependence test developed from Breusch and Pagan (1980) is a useful method. Consider the following panel data regression model:

(5) $$Y_{it}=\alpha_{i}+\beta_{i}X_{it}+\varphi_{it}$$

in which i represents each country in the panel data (i=1,2,…,N), t indicates time (t=1,2,…,T), and $X_{it}$ is a k×1 vector of regressors.

The LM test is illustrated in Eq. (6). The null hypothesis, mathematically expressed as $H_{0}:Cov\left(\varphi_{it},\varphi_{jt}\right)=0$, assumes no cross-sectional dependence. The alternative hypothesis, $H_{1}:\;Cov\left(\varphi_{it},\varphi_{jt}\right)\neq 0$, states the occurrence of cross-sectional dependence.

(6) $$\text{LM}=T\sum_{i=1}^{N-1}\sum_{j=i+1}^{N}{\hat{\rho }}_{ij}$$

Pesaran (2004) provided a modification to the LM test to adjust its bias as follows:

(7) $$\text{CD}=\sqrt{\frac{2T}{N\left(N-1\right)}}\sum_{i=1}^{N-1}\sum_{j=i+1}^{N}\frac{\left(T-k\right){\hat{\rho }}_{ij}^{2}-E\left[\left(T-k\right){\hat{\rho }}_{ij}^{2}\right]}{var\left[\left(T-k\right){\hat{\rho }}_{ij}^{2}\right]}$$

where N is the sample size, T indicates time, and ${\hat{\rho }}_{ij}$ symbolizes the coefficient of pair-wise correlation obtained from OLS estimation of Eq. (5) for each country i (i=1,2,…,N).

#### 3.2.2 Slope homogeneity test

In the presence of cross-sectional dependence, countries in the panel data may interact with each other in an economic-social network, and slope heterogeneity may arise. Thus, it is important to control for the slop heterogeneity so as to avoid unreliable estimation (Breitung, 2005).

To detect slope heterogeneity, Swamy (1970) proposed a pooled estimator to capture the dispersion of the estimated individual regression coefficients, and the null hypothesis of slope homogeneity is tested against the alternative hypothesis of slope heterogeneity. For big panel data, however, Pesaran and Yamagata (2008) developed the Swamy (1970) test to check for slope homogeneity, which is described in Eq. (8):

(8) $$\tilde{\text{S}}=\sum_{i=1}^{N}{\left({\hat{\beta }}_{i}-{\tilde{\beta }}_{WFE}\right)}^{'}\frac{X_{i}^{'}M_{\tau }X_{i}}{{\tilde{\sigma }}_{i}^{2}}\left({\hat{\beta }}_{i}-{\tilde{\beta }}_{WFE}\right)$$

in which ${\hat{\beta }}_{i}$ is the OLS regression coefficient of each country i (i=1,2,…,N), and ${\tilde{\beta }}_{WFE}$ is the weighted fixed effect (WFE) pooled estimator, $M_{\tau }$ indicates the identity matrix, and ${\tilde{\sigma }}_{i}^{2}$ is an estimate of $\sigma_{i}^{2}.\;$ Eqs. (9) and (10) describe the formulas for the standardized dispersion statistic ($\bar{\Delta }$) as well as the biased-adjusted one (${\bar{\Delta }}_{adj}$), which utilizes $\text{E}\left({\bar{z}}_{it}\right)=k\;$ and $\text{var}\left({\bar{z}}_{it}\right)=\frac{2k\left(T-k-1\right)}{T+1}$.

(9) $$\bar{\Delta }=\sqrt{N}\left(\frac{N^{-1}\tilde{\text{S}}-k}{\sqrt{2k}}\right)$$

(10) $${\bar{\Delta }}_{adj}=\sqrt{N}\left(\frac{N^{-1}\tilde{\text{S}}-\text{E}\left({\bar{z}}_{it}\right)}{\sqrt{\text{var}\left({\bar{z}}_{it}\right)}}\right)$$

#### 3.2.3 Panel unit root test

The panel unit root tests that have been developed in the existing literature can be divided into two groups. The first one, also known as the first-generation unit root tests, are instanced as Levin-Lin Chu (LLC), Im-Pesaran-Shin (IPS), augmented Dickey-Fuller (ADF) and Phillips-Perron (PP), and they provide untrustworthy results under cross-sectional dependence. The second one is called second-generation unit root tests which are robust in the presence of cross-sectional dependence (Phillips and Sul, 2003; Pesaran, 2007). Consequently, this article uses two notable second-generation tests including the cross-sectionally augmented Dickey-Fuller (CADF) and the cross-sectionally augmented Im-Pesaran-Shin (CIPS) tests. The CADF statistic is specified in Eqs. (11) and (12):

(11) $$\Delta Y_{i,t}=\alpha_{i}+\beta_{i}Y_{i,t-1}+\gamma_{i}{\bar{Y}}_{t-1}+\delta_{i}\Delta {\bar{Y}}_{i,t}+\varepsilon_{it}$$

(12) $${\bar{Y}}_{t-1}=\frac{1}{N}\sum_{i=1}^{N}Y_{i,t-1};\;\Delta {\bar{Y}}_{i,t}=\frac{1}{N}\sum_{i=1}^{N}\Delta Y_{i,t}\;$$

The CIPS statistics proposed by Pesaran (2007) is calculated from the average of CADF statistics for each country in the panel data (i=1, 2,…,N) computed from the t ratios of $\beta_{i}$ demonstrated in Eq. (11):

(13) $$\text{CIPS}=\frac{1}{N}\sum_{i=1}^{N}CADF_{i}$$

#### 3.2.4 Panel cointegration test

In the presence of cross-sectional dependence, the long-run relationship among variables is inspected by Westerlund panel cointegration test (Westerlund, 2007), which is concluded by detecting whether the error correction exists for individual countries as well as the whole panel. The error correction $\left(\varepsilon_{\text{i}}\right)$ that indicates the speed of correction towards equilibrium is given as:

(14) $$\Delta Y_{i,t}=\delta_{i}^{'}d_{t}+\varepsilon_{i}\left(Y_{i,t-1}-\beta_{i}^{'}X_{i,t-1}\right)+\sum_{j=1}^{p}\varphi_{ij}Y_{i,t-j}+\sum_{j=0}^{p}\varphi_{ij}X_{i,t-j}+\mu_{i,t}$$

Westerlund (2007) employed the group-mean tests (based on $G_{\tau }$ and $G_{\alpha }$ statistics) and the panel tests (based on $P_{\tau }$ and $P_{\alpha }$ statistics) to examine the null hypothesis of no cointegration.

(15) $$G_{\tau }=\frac{1}{N}\sum_{i=1}^{N}\frac{\varepsilon_{i}}{Se\left({\hat{\varepsilon }}_{i}\right)}$$

(16) $$G_{\alpha }=\frac{1}{N}\sum_{i=1}^{N}\frac{T\varepsilon_{i}}{\varepsilon_{i}^{'}}$$

(17) $$P_{\tau }=\frac{{\hat{\varepsilon }}_{i}}{Se\left({\hat{\varepsilon }}_{i}\right)}$$

(18) $$P_{\alpha }=T\hat{\varepsilon }$$

The $G_{\tau }$ and $G_{\alpha }$ statistics are used for detecting whether cointegration manifests itself in at least one cross-sectional country. The $P_{\tau }$ and $P_{\alpha }$ statistics reveal if cointegration appears in the entire panel.

#### 3.2.5 Panel long-run estimates

The cross-sectional dependence phenomenon makes pooled ordinary least squares (OLS) and feasible generalized least squares (GLS) generate biased estimates (Phillips and Sul, 2003). Besides, it prevents common panel models, for instance, fixed effects (FE) and random effects (RE) from achieving consistent and reliable estimates (Sarafidis and Robertson, 2009).

The MG estimator uses OLS method to estimate the time-series regressions of N countries and then average the slope coefficients, which includes heterogeneity in the panel data when the coefficients and error variances can change across countries (Pesaran and Smith, 1995). Nevertheless, it does not contain the common factors in the panel data.

The CCEMG estimator introduced by Pesaran (2006) can not only capture the unobserved common effects ($f_{t}$) but also remain robust in the presence of cross-sectional dependence and slope heterogeneity (Atasoy, 2017; Kapetanios et al., 2011).

(19) $$Y_{it}=\alpha_{i}+\beta_{i}X_{it}+\gamma_{i}{\bar{Y}}_{it}+\delta_{i}{\bar{X}}_{it}+c_{i}f_{t}+\varepsilon_{it}$$

In Eq. (19), $Y_{it}$ is the dependent variable, $X_{it}$ represents the regressors, $\beta_{i}$ denotes the country-specific slope, $f_{t}$ is the unobserved common factor with heterogeneous factor loadings; $\alpha_{i}$ indicates the intercept and $\varepsilon_{it}$ represent the error term.

Another estimator that is also immune to cross-sectional dependence and slope heterogeneity is AMG proposed by Eberhardt and Bond (2009) and Eberhardt and Teal (2010). The AMG estimator employs the common dynamic effect parameter to control for unobservable common factors $f_{t}$, which can give useful interpretations. Eq. (21) provides the formula of the AMG estimator calculated from ${\tilde{\beta }}_{i}$ that are the estimates of $\beta_{i}$ in Eq. (20) which describes an OLS regression at first difference where Δ and θ respectively denote the difference operator and the coefficients of the time dummy D.

(20) $$\Delta Y_{it}=\alpha_{i}+\beta_{i}\Delta X_{it}+\sum_{t=1}^{T}\theta_{t}D_{t}+\varphi_{i}f_{t}+\varepsilon_{it}$$

(21) $$\text{AMG}=\frac{1}{N}\sum_{i=1}^{N}{\tilde{\beta }}_{i}$$

Moreover, the AMG estimator is efficient and unbiased for different N and T settings in Monte Carlo simulation (Bond and Eberhardt, 2013). Hence, this study applies the AMG estimator of Eberhardt and Teal (2010) to assess the long-run parameters. Also, the MG and CCEMG estimators are run alongside for robustness check.

#### 3.2.6 Panel causality tests

Dumitrescu and Hurlin (2012) developed the test for panel data causality from Granger (1969), which is illustrated as follows:

(22) $${\text{Y}}_{\text{i},\text{t}}={\text{α}}_{\text{i}}+\sum_{\text{k}=1}^{\text{P}}{\text{γ}}_{\text{ik}}{\text{Y}}_{\text{i},\text{t}-\text{k}}+\sum_{\text{k}=1}^{\text{P}}{\text{β}}_{\text{ik}}{\text{X}}_{\text{i},\text{t}-\text{k}}+{\text{μ}}_{\text{i},\text{t}}$$

In Eq. (22), $Y_{it}$ and $X_{it}$ are the variables, the subscripts i (i=1,2,…,N) and (t=1,2,…,T) respectively denote country and time, P is the lag length, ${\text{γ}}_{\text{ik}}$ and ${\text{β}}_{\text{ik}}\;$ respectively indicate autoregressive and regression coefficients. Besides, ${\text{β}}_{\text{ik}}\;$ can vary across countries but remains constant over time. In addition, the lag length P is the same for all countries and it must be a positive number. Also, the panel data is supposed to be balanced.

The null hypothesis presumes no causal relationship among the variables for all countries:

(23) $$H_{0}:\;\beta_{i1}=\beta_{i2}\ldots =\beta_{iP}=0,\;\;\forall i=1,\;2,\ldots ,\;N$$

The alternative hypothesis:

(24) $$H_{1}:\;\beta_{i1}=\beta_{i2}\ldots =\beta_{iP}=0,\;\;\forall i=1,\;2,\ldots ,\;\;N_{1}$$

(25) $$\beta_{i1}\neq 0\;or\;\beta_{i2}\neq 0\;,\ldots ,or\;\beta_{iP}\neq 0\;\;\;\forall i=N_{1}\;+1,\;\;N_{1}\;+2,\ldots ,N$$

(Note: $N_{1}$ is a positive integer ranging from 0 to N.)

According to Dumitrescu and Hurlin (2012), the average Wald statistic for the null hypothesis (i.e. no causality in all countries) is obtained from regressing Eq. (22) and conducting F tests for P linear hypotheses $\beta_{i1}=\beta_{i2}\ldots =\beta_{iP}=0$.

(26) $$\bar{W}=\frac{1}{\text{N}}\sum_{\text{i}=1}^{\text{N}}{\text{W}}_{\text{i}}$$

(Note: ${\text{W}}_{i}$ demonstrates the individual country Wald statistics in time T)

Then, from $\bar{W}$, P and N, the standardized Z statistic ($\bar{Z}$) is computed and can be proved to have standard normal distribution when T comes to infinity.

(27) $$\bar{Z}=\sqrt{\frac{N}{2P}}\left(\bar{W}-P\right)\to \text{N}\left(0,1\right)$$

With a given value of T, the harmonized Z test statistic ($\tilde{\text{Z}}$) can be calculated from $\bar{Z}$ and T, and it also follows standard normal distribution (Dumitrescu and Hurlin, 2012).

(28) $$\tilde{\text{Z}}=\sqrt{\frac{N}{2P}X\frac{\left(T-2P-5\right)}{\left(T-P-3\right)}}X\left[\frac{\left(T-2P-3\right)}{\left(T-2P-1\right)}\bar{W}-P\right]\to \text{N}\left(0,1\right)$$

### 3.3 Data

This study evaluates the impacts of energy consumption, government expenditure, institutional quality, financial development and trade openness together with capital on the economic growth of 46 EMDEs (see Appendix) by a multivariate framework with annual data from 1990 to 2014. The real GDP per capita is used as a proxy of economic growth. Capital is represented by the gross fixed capital formation per capita and computed as total amount of gross fixed capital formation divided by the total population. General government final consumption expenditure per capita is measured by the general government final consumption expenditure divided by the total population. The quality of government index is used as a proxy of institutional quality. Energy consumption is measured in kilogram of oil equivalent per capita. In this article, we gauge financial development by 03 components including domestic credit to private sector per capita, domestic credit to private sector by banks per capita and domestic credit provided by financial sector per capita, along with the overall financial development index that summarizes how developed financial institutions and financial markets are in terms of their depth, access, and efficiency. Trade openness per capita is calculated by the total trade value (i.e. export plus import) divided by the total population.

The “per capita” data employed in this article is collected from the World Development Indicators (WDI) database provided by the World Bank, which consists of the real GDP, gross fixed capital formation, government expenditure, energy consumption, domestic credit to private sector, domestic credit to private sector by banks, domestic credit provided by financial sector and trade openness. All aforementioned data except energy use is based on 2010 US$ constant. The data concerning institutional quality is retrieved from The Quality of Government Institute, University of Gothenburg, Sweden, which is gauged by averaging the ICRG variables “Corruption”, “Law and Order” and “Bureaucracy Quality”. It is then normalized to the range [0,1] where the higher value indicates higher institutional quality. The overall financial development index also varies from 0 to 1 and is provided by the International Monetary Fund (IMF). All variables are converted into natural logarithm specification. Table 2 shows information regarding the variables and their sources.

**Table 2:** Variable description and data sources.

Variable name	Symbol	Description	Unit	Data source
Economic growth	GDP	The real gross domestic product	Constant 2010 US dollars, per capita	WDI
Capital	GCF	Gross fixed capital formation	Constant 2010 US dollars, per capita	WDI
Government expenditures	GC	General government final consumption expenditure	Constant 2010 US dollars, per capita	WDI
Institution	QoG	Quality of Government Index. The mean value of the ICRG variables “Corruption”, “Law and Order” and “Bureaucracy Quality”, scaled 0–1. Higher values indicate higher quality of government.	Index (from 0 to 1)	World The Quality of Government Institute, University of Gothenburg, Sweden
Energy consumption	EC	It comprises petroleum products, natural gas, electricity, and combustible renewable and waste.	Kg of oil equivalent per capita	WDI
Financial development (1)	FD1	Domestic credit to private sector	Constant 2010 US dollars, per capita	WDI
Financial development (2)	FD2	Domestic credit to private sector by banks	Constant 2010 US dollars, per capita	WDI
Financial development (3)	FD3	Domestic credit provided by financial sector	Constant 2010 US dollars, per capita	WDI
Financial development Index	FD	It summarizes how developed financial institutions and financial markets are in terms of their depth, access, and efficiency.	Index (from 0 to 1)	IMF
Trade openness	TO	Trade openness measures with export plus import of goods and services	Constant 2010 US dollars, per capita	WDI

## 4 Empirical results

Descriptive statistics including mean, maximum (Max), minimum (Min) and standard deviation (SD) of the variables are given in Table 3 below.

**Table 3:** Descriptive statistics of variables.

Variable	Mean	Max	Min	SD
lnGDP	8.035	10.041	5.087	1.010
lnGCF	6.494	8.915	2.995	1.086
lnGC	5.953	8.831	2.524	1.208
lnQoG	–0.784	–0.216	–2.810	0.298
lnEC	6.670	9.426	4.749	0.793
lnFD1	6.706	9.676	2.638	1.502
lnFD2	6.646	9.628	1.955	1.544
lnFD3	7.010	9.771	1.294	1.470
lnFD	–1.567	–0.349	–3.726	0.594
lnTO	7.542	10.641	4.328	1.261

The multicollinearity among the variables is checked by the correlation matrix displayed in Table 4.

**Table 4:** Correlation matrix of the variables.

	lnGDP	lnGCF	lnGC	lnQoG	lnEC	lnFD1	lnFD2	lnFD3	lnFD	lnTO
lnGDP	1.000									
lnGCF	0.964	1.000								
lnGC	0.937	0.780	1.000							
lnQoG	0.320	0.365	0.383	1.000						
lnEC	0.837	0.796	0.814	0.381	1.000					
lnFD1	0.862	0.806	0.839	0.425	0.771	1.000				
lnFD2	0.839	0.810	0.809	0.425	0.774		1.000			
lnFD3	0.821	0.802	0.783	0.417	0.728			1.000		
lnFD	0.495	0.533	0.475	0.450	0.568				1.000	
lnTO	0.921	0.811	0.800	0.321	0.813	0.840	0.807	0.760	0.450	1.000

It can be observed that energy use, capital, institutional quality, government expenditure, financial development and trade openness are positively correlated with GDP per capita. In addition, the independent variables also positively correlate with each other. The correlation coefficients among the independent variables are quite high, which might cause multicollinearity problem. Hence, in order to evaluate the multicollinearity problem, the author utilizes multicollinearity tests based on VIF (variance inflation factor).^1^ A common cutoff value for VIF is 10 (Hair et al., 1995; Wooldridge, 2013). The results in Table 5 indicate that multicollinearity is likely not a problem when VIF values are smaller than 10.

**Table 5:** Multicollinearity tests based on VIF.

	(1)	(2)	(3)	(4)
lnGCF	8.594	8.422	8.643	8.110
lnGC	7.108	7.044	7.086	7.018
lnQoG	1.268	1.262	1.254	1.326
lnEC	3.566	3.662	3.579	3.854
lnFD1	4.698			
lnFD2		3.936		
lnFD3			3.280	
lnFD				1.715
lnTO	7.867	7.740	7.663	7.816

The estimation procedure begins with cross-sectional dependence CD test provided by Pesaran (2004). It is noticeable in Table 6 that all the p-values associated with real GDP, energy use, gross fixed capital formation, government expenditure, institutional quality, financial development and trade openness variables are statistically significant at 1% level, which evidences the occurrence of cross-sectional dependence the panel data.

**Table 6:** Results of cross-sectional dependence test.

Variable	CD-test	P-value
lnGDP	110.621∗∗∗	0.000
lnGCF	78.844∗∗∗	0.000
lnGC	60.519∗∗∗	0.000
lnQoG	30.683∗∗∗	0.000
lnEC	64.423∗∗∗	0.000
lnFD1	70.768∗∗∗	0.000
lnFD2	70.998∗∗∗	0.000
lnFD3	58.012∗∗∗	0.000
lnFD	75.766∗∗∗	0.000
lnTO	94.956∗∗∗	0.000

Notes: ∗∗∗ indicates significance at 1% level. The null hypothesis is no cross-sectional dependence.

Next, slope heterogeneity is checked by Pesaran and Yamagata (2008) test. From the results in Table 7 where all standardized dispersion statistics ($\bar{\Delta }$) and biased-adjusted statistics (${\bar{\Delta }}_{adj}$) are statistically significant at 1% level, we can conclude the existence of slope heterogeneity in the panel data.

**Table 7:** Slope homogeneity test results.

Variable	$\bar{\Delta }$	${\bar{\Delta }}_{adj}$
lnGDP	79.39∗∗∗	153.05∗∗∗
lnGCF	87.39∗∗∗	166.51∗∗∗
lnGC	167.98∗∗∗	437.37∗∗∗
lnQoG	68.06∗∗∗	111.48∗∗∗
lnEC	518.60∗∗∗	589.71∗∗∗
lnFD1	271.31∗∗∗	377.54∗∗∗
lnFD2	259.58∗∗∗	395.19∗∗∗
lnFD3	114.36∗∗∗	129.33∗∗∗
lnFD	173.50∗∗∗	367.35∗∗∗
lnTO	217.24∗∗∗	280.72∗∗∗

Notes: ∗∗∗ indicates significance at 1% level. The null hypothesis is slope homogeneity (no heterogeneity).

Now, owing to presence of cross-sectional dependence and slope heterogeneity, we use second-generation CADF and CIPS unit root tests (Pesaran, 2007) to investigate the stationarity of the variables. The outcomes in Table 8 demonstrated that all variables are integrated at order 1 (i.e. stationary at first difference). Thus, there are only I (1) variables in the panel data.

**Table 8:** Panel unit root tests results.

Variables	CADF test Statistic		CIPS test Statistic	
	Level	First difference	Level	First difference
lnGDP	–1.917	–3.473∗∗∗	–1.928	–3.186∗∗∗
lnGCF	–1.438	–2.735∗∗∗	–1.394	–3.039∗∗∗
lnGC	–1.473	–2.764∗∗∗	–1.381	–3.026∗∗∗
lnQoG	–1.378	–2.693∗∗∗	–1.404	–3.842∗∗∗
lnEC	–1.651	–2.856∗∗∗	–1.821	–4.035∗∗∗
lnFD1	–1.398	–2.773∗∗∗	–1.477	–3.215∗∗∗
lnFD2	–1.337	–2.756∗∗∗	–1.491	–3.196∗∗∗
lnFD3	–1.076	–2.671∗∗∗	–1.185	–3.082∗∗∗
lnFD	–1.252	–2.693∗∗∗	–1.329	–3.114∗∗∗
lnTO	–0.990	–2.175∗∗	–1.498	–3.779∗∗∗

Notes: ∗∗∗ indicates significance at 1% level. The null hypothesis is non-stationarity.

Next, as cross-sectional dependence and slope heterogeneity appear in the panel data and all the variables are integrated at order 1, we utilize Westerlund (2007) cointegration test to inspect the long-run relationship among the variables. All robust p-values in Table 9 are statistically significant below 5% level, thus rejecting the null hypothesis and confirming the cointegration among real GDP, energy use, gross fixed capital formation, government expenditure, institutional quality, financial development and trade openness.

**Table 9:** Westerlund (2007) cointegration test results.

Statistics	Value	Z-value	Robust P-value
$G_{\tau }$	–2.927∗∗∗	–6.094	0.000
$G_{\alpha }$	–11.151∗∗	2.990	0.017
$P_{\tau }$	–23.916∗∗∗	–6.014	0.000
$P_{\alpha }$	–12.525∗∗∗	–5.175	0.003

Notes: ∗∗ and ∗∗∗ respectively indicate significance at 5% and 1% levels. The null hypothesis is no cointegration.

For estimations of long-run parameters, this paper employs the estimators that work well under cross-sectional dependence and slope heterogeneity including AMG (Bond and Eberhardt, 2013; Eberhardt and Teal, 2010), MG (Pesaran and Smith, 1995) and CCEMG (Pesaran, 2006) to evaluate the long-run parameters. In addition, the AMG estimator is used as the main approach while the other ones are used for robustness check. We also include 4 proxies for financial development (i.e. domestic credit to private sector per capita, domestic credit to private sector by banks per capita, domestic credit provided by financial sector per capita and financial development index) in the models displayed in Table 10.

**Table 10:** Heterogeneous parameter estimates using AMG, MG, and CCEMG estimators.

Regressors	(1)			(2)			(3)			(4)		
	AMG	CCEMG	MG	AMG	CCEMG	MG	AMG	CCEMG	MG	AMG	CCEMG	MG
lnGCF	0.128∗∗∗	0.122∗∗∗	0.128∗∗∗	0.127∗∗∗	0.108∗∗∗	0.132∗∗∗	0.134∗∗∗	0.121∗∗∗	0.126∗∗∗	0.129∗∗∗	0.118∗∗∗	0.140∗∗∗
	(7.51)	(6.6)	(7.21)	(7.54)	(5.6)	(8.01)	(8.04)	(8.29)	(6.44)	(7.41)	(7.69)	(7.33)
lnGC	0.101∗∗∗	0.052∗∗∗	0.185∗∗∗	0.098∗∗∗	0.047∗∗∗	0.187∗∗∗	0.132∗∗∗	0.093∗∗∗	0.203∗∗∗	0.125∗∗∗	0.070∗∗∗	0.067∗∗∗
	(5.10)	(3.88)	(5.03)	(4.95)	(2.96)	(5.09)	(5.39)	(4.7)	(5.4)	(6.32)	(4.24)	(3.61)
lnQoG	0.019	0.011	0.003	0.016	0.005	0.002	0.030	0.031	0.017	0.022	0.008	0.008
	(0.84)	(0.78)	(0.09)	(0.74)	(0.4)	(0.06)	(1.17)	(1.52)	(0.55)	(1.05)	(0.4)	(0.49)
lnEC	0.177∗∗∗	0.097∗∗∗	0.210∗∗	0.173∗∗∗	0.103∗∗∗	0.227∗∗∗	0.171∗∗∗	0.162∗∗∗	0.213∗∗	0.192∗∗∗	0.116∗∗∗	0.154∗∗∗
	(3.73)	(3.74)	(2.22)	(3.59)	(3.63)	(2.58)	(4.16)	(5.59)	(2.35)	(4.58)	(4.4)	(4.28)
lnFD1	0.019∗∗∗	0.016∗∗∗	0.054∗∗∗									
	(4.84)	(4.82)	(2.71)									
lnFD2				0.022∗∗∗	0.022∗∗	0.050∗∗∗						
				(2.80)	(2.31)	(2.59)						
lnFD3							0.026∗∗	0.034∗∗	0.049∗∗			
							(2.05)	(2.31)	(2.08)			
lnFD										0.016∗∗	0.010∗∗	0.022∗∗
										(2.03)	(2.00)	(2.54)
lnTO	0.032∗∗	0.038∗∗	0.108∗∗∗	0.029∗∗	0.046∗∗	0.109∗∗∗	0.042∗∗	0.039∗∗	0.103∗∗∗	0.046∗∗∗	0.049∗∗∗	0.026∗∗∗
	(2.06)	(2.12)	(4.84)	(1.96)	(2.51)	(4.87)	(2.33)	(1.97)	(4.38)	(3.00)	(3.15)	(5.97)

Notes: t-statistics are in parentheses. ∗∗, ∗∗∗ indicate significance at the 5% and 1% significance levels respectively.

As AMG estimator is the main method used in this article, we will list some remarkable coefficients estimated by it in the 4 models (1), (2), (3) and (4). Specifically, 1% increase of EC (energy consumption per capita) improves GDP per capita by between 0.171% and 0.192%. Also, 1% growth of GCF (gross fixed capital formation per capita) raises GDP per capita by between 0.127% and 0.134%. Next, 1% rise of GC (general government final consumption expenditure per capita) boosts GDP per capita by between 0.098% and 0.132%. Meanwhile, 1% improvement of TO (trade openness) fosters GDP per capita by between 0.029% and 0.046%.

Concerning the effects of financial development, 1% increase of FD1 (domestic credit to private sector per capita) facilitates GDP per capita by 0.019%. In addition, when FD2 (domestic credit to private sector by banks per capita), FD3 (domestic credit provided by financial sector per capita) and FD (financial development index) each grow by 1%, the GDP per capita is enhanced by 0.022%, 0.026% and 0.016% respectively.

From Table 10, it is notable that all coefficient signs in all models estimated by the three estimators are the same, which affirms the consistency and robustness of the empirical results.

After the long-run estimation of panel data, now it is time to inspect the causality among the variables by Dumitrescu and Hurlin (2012) test to ensure robustness. The results listed in Table 11.

**Table 11:** Results of Dumitrescu and Hurlin (2012) Granger causality test results.

Variables	lnGDP	lnGCF	lnGC	lnQoG	lnEC	lnTO	lnFD1	lnFD2	lnFD3	lnFD
lnGDP	–	4.36∗∗∗	10.45∗∗∗	10.25∗∗∗	3.48∗∗∗	3.49∗∗∗	4.24∗∗∗	3.82∗∗∗	4.15∗∗∗	3.98∗∗∗
		(7.99)	(11.7)	(11.26)	(5.02)	(11.92)	(7.58)	(13.55)	(7.28)	(6.72)
lnGCF	6.38∗∗∗	–	3.52∗∗∗	1.51∗∗	4.60∗∗∗	14.62∗∗∗	10.49∗∗∗	2.61∗∗∗	2.19∗∗∗	2.03∗∗∗
	(14.84)		(12.07)	(2.44)	(8.82)	(20.64)	(11.78)	(7.71)	(5.71)	(4.91)
lnGC	7.89∗∗∗	5.09∗∗∗	–	10.16∗∗∗	4.35∗∗∗	4.31∗∗∗	12.34∗∗∗	12.80∗∗∗	3.15∗∗∗	4.72∗∗∗
	(33.04)	(19.63)		(11.06)	(16.05)	(15.89)	(15.74)	(16.74)	(10.33)	(9.22)
lnQoG	4.11∗∗∗	7.87∗∗∗	5.12∗∗∗	–	3.93∗∗∗	8.54∗∗∗	10.37∗∗∗	9.99∗∗∗	2.45∗∗∗	4.44∗∗∗
	(7.15)	(6.15)	(10.59)		(6.54)	(7.59)	(11.53)	(10.71)	(6.94)	(8.27)
lnEC	11.17∗∗∗	2.79∗∗∗	3.94∗∗∗	2.32∗∗∗	–	3.52∗∗∗	2.77∗∗∗	2.86∗∗∗	2.23∗∗∗	2.18∗∗∗
	(13.23)	(8.58)	(6.57)	(6.35)		(12.07)	(8.48)	(8.9)	(5.88)	(5.65)
lnTO	2.81∗∗∗	1.94∗∗∗	2.57∗∗∗	1.31	2.19∗∗∗	–	1.61∗∗∗	1.55∗∗∗	3.43∗∗∗	2.42∗∗∗
	(8.7)	(4.5)	(7.55)	(1.49)	(5.72)		(2.91)	(2.62)	(11.65)	(6.8)
lnFD1	11.05∗∗∗	6.44∗∗∗	16.39∗∗∗	9.76∗∗∗	12.23∗∗∗	5.92∗∗∗				
	(30.68)	(15.06)	(24.44)	(10.21)	(15.51)	(13.31)				
lnFD2	10.22∗∗∗	9.22∗∗∗	15.73∗∗∗	9.49∗∗∗	12.80∗∗∗	9.66∗∗∗				
	(27.87)	(9.05)	(23.01)	(9.64)	(16.72)	(10)				
lnFD3	10.22∗∗∗	5.48∗∗∗	3.49∗∗∗	3.04∗∗∗	3.21∗∗∗	5.72∗∗∗				
	(27.87)	(21.46)	(11.93)	(9.79)	(10.57)	(12.63)				
lnFD	5.29∗∗∗	8.57∗∗∗	11.10∗∗∗	10.05∗∗∗	3.76∗∗∗	3.55∗∗∗				
	(20.6)	(7.66)	(13.08)	(10.84)	(13.26)	(12.24)				

Notes: The given numbers are W statistics. Z statistics are shown in parentheses. ∗∗∗ indicates significance at 1% level. Y→X indicates the null hypothesis that X does not cause Y.

In Table 11, the feedback effects between economic growth and the other factors are validated, as evidenced by the statistically significant W statistics in any pair of variables containing GDP. In other words, gross fixed capital formation, government expenditures, institution, energy consumption, financial development and trade openness Granger-cause economic growth. Also, economic growth Granger-causes the other factors. This indicates that the economic growth of EMDEs depends on finance and investment, energy consumption, international trade as well as the role of government in government expenditures and institutional improvement.

## 5 Concluding remarks and recommendations

This study examines the impacts of energy consumption, government expenditure, institutional quality, financial development and trade openness together with capital on the economic growth of 46 EMDEs by a multivariate framework using annual data from 1990 to 2014. We apply CADF and CIPS unit root tests to investigate the stationary properties of the variables after detecting cross-sectional dependence and slope heterogeneity in the panel data. We also find the evidence for long-run relationship among energy use, gross fixed capital formation, institutional quality, government expenditure, financial development, trade openness and economic growth in EMDEs by Westerlund cointegration test. The long-run effects are estimated by second-generation estimators including Augmented Mean Group (AMG), Mean Group (MG), and Common Correlated Effects Mean Group (CCEMG). The causation among the variables are inspected by Dumitrescu and Hurlin causality analysis.

The findings indicate that energy use, gross fixed capital formation, government expenditure, financial development and trade openness positively affect the economic growth of EMDEs. Moreover, the feedback hypothesis is validated in the connection between energy use and economic growth. In addition, the feedback effect also exists in the association between the other variables and economic growth.

From the empirical results, policy-makers in EMDEs should implement policies that foster financial development, thus facilitating economic growth. This necessitates appropriate fiscal policies through government expenditure as well as active and flexible government intervention in monetary policies in order to maintain stable macroeconomic environment. Policies that relax credit restriction should be encouraged to reduce capital expenses as well as allocate financial resources effectively. Besides, the findings also support the trade-led growth hypothesis in EMDEs. Thus, so as to fully exploit the effectiveness of trade-led growth policies, EMDEs countries should encourage and attract foreign direct investment as well as increase the investment and the size of the exportation sector. It is more likely for the trade policies to be successful if they are combined with the development of the financial sector that lower capital restriction.

Concerning energy consumption, the empirical results of this study disclose that energy consumption can stimulate the economic growth in EMDEs. Meanwhile, economic growth also causes more energy consumption. This possibly implies the trade-off between economic growth and environmental quality in EMDEs. Consequently, limiting energy consumption will exacerbate the economic growth of EMDEs when energy is one of the vital factors contributing to the growth of these countries. Rather, EMDEs should consider policies that promote efficient energy use, upgrade obsolete technology to modern and efficient one and support the research, investment and application of renewable energy so as to reduce the detrimental impacts on the environment as well as ensure sufficient energy for economic development. Last but not least, EMDEs should strengthen the institutional quality reform through transparency in governance, corruption control, and the improvement of the legal system.

## Declarations

### Author contribution statement

Hoang Phong Le: Conceived and designed the experiments; performed the experiments; analyzed and interpreted the data; contributed reagents, materials, analysis tools or data; wrote the paper.

### Funding statement

This work was supported by the European Union's Horizon 2020 research and innovation programme under Marie Skłodowska-Curie grant agreement No. 734712. This work was also supported by the University of Economics Ho Chi Minh City.

### Competing interest statement

The authors declare no conflict of interest.

### Additional information

No additional information is available for this paper.

## Footnotes

1 The author thanks the reviewer for the notes about multicollinearity.
