# Supplementary material for: The energy-growth nexus revisited: the role of financial development, institutions, government expenditure and trade openness
Source: Heliyon. 2020 Jul 14;6(7):e04369. doi: 10.1016/j.heliyon.2020.e04369 (PMC7364027; doi:10.1016/j.heliyon.2020.e04369)
Supplement: Appendix [file mmc1.docx]

**Appendix.** List of 46 Emerging Market and Developing Economies

| No. | Country | No. | Country | No. | Country | No. | Country | No. | Country |
| --- | --- | --- | --- | --- | --- | --- | --- | --- | --- |
| 1 | Algeria | 11 | Colombia | 21 | Guatemala | 31 | Nicaragua | 41 | Sudan |
| 2 | Argentina | 12 | Congo, Rep. | 22 | India | 32 | Nigeria | 42 | Tanzania |
| 3 | Bahrain | 13 | Costa Rica | 23 | Indonesia | 33 | Oman | 43 | Thailand |
| 4 | Bangladesh | 14 | Cote d'Ivoire | 24 | Jordan | 34 | Pakistan | 44 | Tunisia |
| 5 | Bolivia | 15 | Dominican Republic | 25 | Kenya | 35 | Panama | 45 | Turkey |
| 6 | Botswana | 16 | Ecuador | 26 | Lebanon | 36 | Paraguay | 46 | Uruguay |
| 7 | Brazil | 17 | Egypt, Arab Rep. | 27 | Malaysia | 37 | Peru |  |  |
| 8 | Cameroon | 18 | El Salvador | 28 | Mexico | 38 | Philippines |  |  |
| 9 | Chile | 19 | Gabon | 29 | Morocco | 39 | Saudi Arabia |  |  |
| 10 | China | 20 | Ghana | 30 | Mozambique | 40 | Senegal |  |  |
